# Patient’s travel distance to specialised cancer diagnostics and the association with the general practitioner’s diagnostic strategy and satisfaction with the access to diagnostic procedures: an observational study in Denmark

**DOI:** 10.1186/s12875-020-01169-y

**Published:** 2020-05-31

**Authors:** Line Flytkjær Virgilsen, Line Hvidberg, Peter Vedsted

**Affiliations:** 1 Research Unit for General Practice, Aarhus, Bartholins Allé 2, 8000 Aarhus C, Denmark; 2 Research Centre for Cancer Diagnosis in Primary Care (CaP), Department of Public Health, Aarhus University, Bartholins Allé 2, 8000 Aarhus C, Denmark; 3 Department of Quality and Improvement, Hospital of South West Jutland, Finsensgade 35, 6700 Esbjerg, Denmark

**Keywords:** General practice, Travel distance, Diagnostic strategy, GP satisfaction, Cancer, Early diagnosis, Denmark

## Abstract

**Background:**

Research indicate that when general practitioners (GPs) refer their patients for specialist care, the patient often has long distance. This study had a twofold aim: in accordance to the GP’s suspicion of cancer, we investigated the association between: 1) cancer patient’s travel distance to the first specialised diagnostic facility and the GP’s diagnostic strategy and 2) cancer patient’s travel distance to the first specialised diagnostic facility and satisfaction with the waiting time and the availability of diagnostic investigations.

**Method:**

This combined questionnaire- and registry-based study included incident cancer patients diagnosed in the last 6 months of 2016 where the GP had been involved in the diagnostic process of the patients prior to their diagnosis of cancer (*n* = 3455). The patient’s travel distance to the first specialised diagnostic facility was calculated by ArcGIS Network Analyst. The diagnostic strategy, cancer suspicion and the GP’s satisfaction with the waiting times and the available investigations were assessed from GP questionnaires.

**Results:**

When the GP did not suspect cancer or serious illness, an insignificant tendency was seen that longer travel distance to the first specialised diagnostic facility increased the likelihood of the GP using ‘wait-and-see’ approach and ‘medical treatment’ as diagnostic strategies. The GPs of patients with travel distance longer than 49 km to the first specialised diagnostic facility were more likely to report dissatisfaction with the waiting time for requested diagnostic investigations (PR: 1.98, 95% CI: 1.20–3.28).

**Conclusion:**

A insignificant tendency to use ‘wait-and-see’ and ‘medical treatment’ were seen among GPs of patients with long travel distance to the first diagnostic facility when the GP did not suspect cancer or serious illness. Long distance was associated with higher probability of GP dissatisfaction with the waiting time for diagnostic investigations.

## Background

Western healthcare systems have increasingly centralised and specialised healthcare services over the recent decades [1, 2]. Travel distance to medical consultations and accessibility of GPs are central topics in many healthcare systems due to shortage of GPs and increasing centralisation. Further, in countries with a gatekeeping system, GPs may have to consider long distances when referring patients to specialised care [1–5].

The literature is inconclusive on the potential impact of distance to diagnostic services on cancer outcomes. Some studies have suggested that longer distance to healthcare services or rural residence are associated with poorer cancer prognosis [6–9]. Others have reported better prognosis in patients with long distance to healthcare services or rural residence [10, 11]. Some studies have found no association [12, 13]. Our research group has found that the association between distance to cancer diagnostics and diagnostic delays in the cancer trajectory depends on the diagnostic difficulty of the underlying cancer [14]. Other studies, including a few qualitative studies, have attempted to establish the direction and the magnitude of the impact of distance on cancer outcomes [15, 16], but possible explanations remain understudied.

GPs play a crucial role in early cancer detection because the majority of cancer patients initially consult their GP with symptoms [17, 18]. In approximately 50% of patients who begin the diagnostic journey in general practice, the GP will suspect cancer at the first presentation [19], and the suspicion is more often raised when the patient present with alarm symptoms [19, 20]. The GP often has a range of strategies available for further relevant diagnostics including in-house options as “wait-and-see”, blood tests and medical treatment such as prescription medicine. The GP can also act as a gatekeeper to e.g. specialised private practices, Cancer Patient Pathways (CPP) or diagnostic imaging (e.g. CT scan). Therefore, the actions taken by the GP upon the patient’s symptom presentation may considerably affect the cancer trajectory [17]. Nevertheless, it is unknown if the GP’s diagnostic strategy is affected by the patient’s travel distance to the medical facility performing cancer diagnostic investigations. Furthermore, we do not know whether this travel distance may affect the GP’s satisfaction with the access to and waiting time for diagnostic investigations.

The aim of this study was twofold: in accordance to the GP’s suspicion of cancer, we investigate the association between 1) cancer patient’s travel distance to the first specialised diagnostic facility and the GP’s diagnostic strategy and 2) cancer patient’s travel distance to the first specialised diagnostic facility satisfaction with the waiting time and the availability of diagnostic investigations.

## Methods

### Study design and setting

This nationwide observational study based on combined questionnaire- and registry data was conducted in Denmark with a population of 5.8 million in 2018. The healthcare system is tax-funded and offers free access to healthcare for all citizens. The GP acts as a gatekeeper serving as the first point of contact to the healthcare system, and 99% of the Danish population is registered with a general practice, which must be consulted for medical advice [21]. GPs can refer patients to investigations and treatment at hospitals and private practicing specialists. CPPs have been implemented for more than 30 types of cancer to ensure standardised national guidelines on the diagnostics and treatment of cancer [22].

### Participants

The study population was defined as patients aged 30–99 years recorded with an incident cancer diagnosis (excluding ICD-10: C44) between 1 July and 20 December 2016 in the Danish National Patient Register (NPR) [23].

Patients were eligible if a first registered cancer related investigation could be assessed in Danish nationwide registers and two data sources were used. First, patients undergoing a first diagnostic investigation at a specialised private practice (gynaecology, ear-nose-throat specialist, eye specialist or dermatologist) were identified in the National Health Insurance Service Register (NHISR) (specialty codes: 04, 07, 15, 16, 19, 21, 39, 41). These specialties serve as filter functions in a number of CPPs often referred by a GP. Contacts up to 3 months prior to diagnosis were assessed in the NHISR, and the first registered contact in this period was selected. Second, patients for whom no diagnostic investigations had been recorded in the NHISR were identified through hospital contacts recorded in the NPR [23]. We identified contacts to the hospital for up to 3 months prior to the date of diagnosis and selected the first relevant contact (an ICD-10 DC or DZ code, excluding Z08-Z09, Z20-Z29, Z30-Z39, Z55-Z65, Z70-Z76).

The GPs of the included patients received a questionnaire between 28 April 2017 and 10 January 2018 for each patient who had given consent to contact their GP. If the patient had deceased shortly after the diagnosis, permission to contact was granted by the Danish Patient Safety Authority [24]. As the cancer was diagnosed up to 1½ year before the GP received the questionnaire, it was emphasised that the GP should fill in the questionnaire based on the medical records. The questionnaire focussed on the following themes: milestone dates in the cancer trajectory, diagnostic strategy, routes to diagnosis and satisfaction with diagnostic procedures [25]. A GP could fill in questionnaires for more than one patient.

The inclusion criteria were: 1) the GP had completed the questionnaire, 2) the GP was involved in the diagnostic process. Thus, GP responses of 3455 patients were eligible for inclusion in the study.

### Data sources

All data sources were linked through the patient’s unique civil registration number (CRN), which is allocated to all Danish residents and used at every contact with the healthcare system [26]. Information on the GP’s diagnostic strategy and level of satisfaction with diagnostic investigations was obtained from the GP questionnaire.

### Variables

*Diagnostic strategy* of the GP was assessed from the GP questionnaire where the GP was asked, “Which actions did you/your practice take in the time from when the patient first contacted your practice until you/your practice referred the patient for further investigations for the first time?” The following response options were included in the questionnaire as an option: 1) *wait-and-see approach* (yes/no), 2) *medical treatment* (yes/no) and 3) *referral to further diagnostic investigation on the same day* (yes/no). Referral can either be to a CPP, diagnostic unit, specialised private practice, diagnostic imaging or other laboratory test at the hospital. For this study, referral was investigated in general terms and not distinguished between different referral modalities as this depend on e.g. the patients symptoms and the GPs options for referral.

*Satisfaction with waiting time and available diagnostic investigations* was assessed from the two following questions in the GP questionnaire: “How satisfied were you with the availability of diagnostic investigations?” and “How satisfied were you with the waiting time for diagnostic investigations?” The response categories “very satisfied” and “satisfied” were combined into “satisfied”, and “dissatisfied” and “very dissatisfied” were combined into “dissatisfied”. The response category “do not know/not relevant” was omitted. The availability of diagnostic investigations vary across regions in Denmark and the GP answered these questions based on his or her availability.

*Travel distance* (shortest road distance) between patient’s residential address on the date of diagnosis and the diagnostic facility where the first diagnostic investigation took place in the cancer trajectory was calculated by ArcGIS Network Analyst [27]. This information was obtained from the Danish Civil Registration System [26]. Distance to the first diagnostic facility was calculated for 3231 patients (first contact to a hospital: 87%; first contact to a specialised private practice: 13%). To avoid possible outliers or inclusion of erroneous registrations, it was chosen to exclude patients with a distance of more than 100 km to the first diagnostic facility (*n* = 76), as it is unlikely in Denmark that the first relevant diagnostic investigation is so far from the patients residence. Thus, 3155 patients were included in the analysis.

### Confounders

The following variables were included as potential confounders based on data from Statistics Denmark: age, sex, patient’s education categorised according to UNESCO’s International Standard Classification of Education [28] (low: ≤10 years, middle: > 10 ≤ 15 years and high: > 15 years) and patient’s marital status (married/cohabiting or living alone). Information on cancer type was obtained from the NPR and included as a potential confounder categorised into: 1) breast cancer, 2) gynaecological cancer, 3) cancer in male genitals, 4) cancer in the digestive organs, 5) cancer in the respiratory system, 6) malignant melanoma, 7) haematological cancer or lymphomas and 8) other types of cancer.

### Statistical analysis

Generalised linear models (GLMs) with prevalence ratios (PRs) and 95% confidence intervals (CI) were applied to study the association between travel distance from patient’s residence to the first specialised diagnostic facility and the GP’s diagnostic strategy and satisfaction with availability for diagnostic investigations. Distance to the medical facility performing the first specialised diagnostic investigation was categorised into the 25, 50, 75 and 90% centiles, corresponding to 0–6 km, > 6–18 km, > 18–34 km, > 34–49 km and > 49 km.

As the association between travel distance to the first diagnostic facility and the GP’s diagnostic strategy interacted with the GP’s suspicion of cancer, these analyses were presented stratified based on whether the GP suspected cancer or not.

For both the analysis on the GP’s diagnostic strategy and on satisfaction with the waiting time, the results were presented unadjusted followed by a model adjusting for patient’s sex, age, education, marital status and cancer type. Prior to this, it was tested if distance was associated with the GPs suspicion of cancer or serious illness, which was not the case.

Stata statistical software, release 15.0, was used for all analyses.

## Results

### Participants and descriptive data

Among the 3155 included patients, the median age was 70 years, the majority were male, had middle-level education and were married (Table 1). The GP suspected cancer or serious illness in 64% of the patients at first presentation. Half of the patients lived within 18 km of the medical facility performing their first specialised diagnostic investigation (Table 1).

**Table 1:** Socio-economic position and patient’s travel distance to first specialised cancer facility in the study population (numbers vary due to missing data)

	N	(%)
**Total**	3155	(100)
**Age, median (IQR)**	70 (61–76)	
**Sex**		
Female	1507	(47.8)
Male	1648	(52.2)
**Education**		
Low	996	(32.4)
Middle	1399	(45.5)
High	678	(22.1)
**Marital status**		
Married/cohabiting	1976	(63.4)
Living alone	1143	(36.6)
**GP’s suspicion of cancer or serious illness**		
No	1084	(35.0)
Yes	2013	(64.0)
**Cancer type**		
Breast cancer	448	(14.2)
Gynaecological cancer	165	(5.2)
Cancer in the male genitals	491	(15.6)
Cancer in the digestive system	726	(23.0)
Cancer in the respiratory system	443	(14.0)
Haematological cancer and lymphomas	227	(7.2)
Malignant melanoma	237	(7.5)
Others	418	(13.3)
**Distance**^**a**^**(km), median (IQR)**	18 (6–34)	
**Distance**^**a**^**(km) categorical**		
0–6	785	(25.0)
> 6–18	778	(24.7)
> 18–34	778	(24.7)
> 34–49	493	(15.6)
> 49	321	(10.2)

^a^Travel distance from the residence of the patient to the patient’s first specialised cancer facility

### Outcome: patient’s travel distance and GP’s diagnostic strategy

When the GP did not suspect cancer, there was an insignificant tendency that travel distance of more than 6 km to the patient’s first diagnostic cancer investigation was associated with higher likelihood of the GP selecting ‘wait-and-see’ and ‘medical treatment’ as diagnostic strategy. However, this was only statistically significant in the distance category > 6–18 km for ‘wait-and-see’ and in the category > 18–34 km for ‘medical treatment’ (Table 2).

**Table 2:** The association between patient’s travel distance to first specialised diagnostic facility and the probability of the GP using “wait-and-see”, “medical treatment” and “referral the same day”(i.e. the diagnostic strategy of the GP) stratified on whether the GP suspected cancer or serious illness or not

	Wait-and-see^**b**^		Medical treatment^**b**^		Referred the same day^**b**^	
	GP did suspect cancer or serious illness (***n*** = 2013)	GP did ***not*** suspect cancer or serious illness (***n*** = 1084)	GP did suspect cancer or serious illness (***n*** = 2013)	GP did ***not*** suspect cancer or serious illness (***n*** = 1084)	GP did suspect cancer or serious illness (***n*** = 2013)	GP did ***not*** suspect cancer or serious illness (***n*** = 1084)
	PR_adj_ (95%CI)	PR_adj_ (95%CI)	PR _adj_ (95%CI)	PR_adj_ (95%CI)	PR_adj_ (95%CI)	PR_adj_ (95%CI)
**Distance**^**a**^**(km)**						
0–6	1 (ref.)	1 (ref.)	1 (ref.)	1 (ref.)	1 (ref.)	1 (ref.)
> 6–18	0.95 (0.45–1.91)	**1.48 (1.08–2.04)**	0.92 (0.51–1.64)	1.25 (0.91–1.72)	0.97 (0.90–1.04)	0.82 (0.63–1.08)
> 18–34	0.84 (0.42–1.67)	1.15 (0.82–1.61)	0.88 (0.47–1.58)	**1.45 (1.05–1.99)**	0.99 (0.92–1.06)	0.84 (0.64–1.12)
> 34–49	0.44 (0.16–1.19)	1.19 (0.84–1.70)	1.09 (0.58–2.04)	1.29 (0.89–1.87)	1.04 (0.97–1.12)	0.86 (0.62–1.19)
> 49	1.21 (0.52–2.79)	1.21 (0.81–1.82)	1.05 (0.50–2.21)	1.29 (0.88–1.91)	1.01 (0.92–1.11)	1.07 (0.78–1.46)

Analysis were based on 3097 patients, due to missing responses regarding GP’s suspicion of cancer

^a^Travel distance from the residence of the patient to the first specialised facility

^b^Adjusted for sex, age (continuous), education, marital status and cancer type

### Outcome: patient’s travel distance and GP’s satisfaction with investigations

GPs of patients with a distance of more than 49 km to the first diagnostic facility were more likely to report dissatisfaction with the waiting time for diagnostic investigations (PR_adj_ 1.98, 95% CI: 1.20–3.28). There was also a not statistically significant tendency that GPs of patients with a distance of more than 49 km to the first diagnostic facility were more likely to report dissatisfaction with the availability of diagnostic investigations (Table 3).

**Table 3:** The association between patient’s travel distance to the first diagnostic facility and the probability of the GP being dissatisfied with diagnostic investigations

	GP’s dissatisfaction with:					
	Availability of diagnostic investigations			Waiting time for diagnostic investigations		
	N_dissatisfied_	PR_unadj_	PR_adj_^b^	N_dissatisfied_	PR_unadj_	PR_adj_^b^
**Distance**^**a**^**(km)**						
0–6	17	1 (ref.)	1 (ref.)	33	1 (ref.)	1 (ref.)
> 6–18	15	0.89 (0.45–1.77)	0.91 (0.46–1.80)	43	1.33 (0.86–2.07)	1.33 (0.85–2.08)
> 18–34	9	0.52 (0.23–1.16)	0.58 (0.26–1.30)	28	0.87 (0.53–1.42)	0.90 (0.55–1.49)
> 34–49	8	0.74 (0.32–1.70)	0.89 (0.38–2.06)	21	1.00 (0.59–1.71)	1.07 (0.62–1.84)
> 49	12	1.72 (0.83–3.56)	1.89 (0.90–3.85)	24	**1.86 (1.12–3.09)**	**1.98 (1.20–3.28)**

^a^ Travel distance from the residence of the patient to the first specialised investigation

^b^Adjusted for sex, age (continuous), education, marital status, cancer type and cancer type

## Discussion

### Key results

When GPs did not suspect cancer or serious illness at first presentation, there was an insignificant tendency that patient’s travel distance to the first diagnostic cancer investigation was associated with the GP using ‘wait-and-see’ or ‘medical treatment’ as initial diagnostic strategy. Furthermore, when the patient’s travel distance to the first specialised diagnostic facility exceeded 49 km, lower GP satisfaction was seen with the waiting time for GP-requested diagnostic investigations.

### Strengths and limitations

The assessment of the GP’s diagnostic strategy and the level of satisfaction was based on responses from a national questionnaire survey among GPs of newly diagnosed patients, and the identification of patients was based on the NPR, which is known to have valid and complete data [23]. The GPs were asked to check their medical records when responding to the questionnaire. Hence, even though the questionnaire was answered in retrospect and some 1½ years after the cancer diagnosis was given, the risk of recall bias was minimised. The inclusion of GPs was determined by patients approving the inclusion of their GP in the study, except for GPs with patients who had deceased shortly after the diagnosis. It is likely that non-responding patients were more likely to have lower socio-economic status (SEP). GPs of patients with low SEP might thus have been be less representative in the study. Still, this has been shown not to bias outcome estimates markedly [29].

We used valid and complete registers to obtain information on SEP and cancer type of the patients [30]. The first diagnostic facility was also based on registry information from hospitals [23] and specialised private practices [31]; this information was identified in the registers based on an algorithm assessing contacts in the healthcare system 3 months prior to the diagnosis. Misclassification cannot be ruled out as the first diagnostic investigation might have been performed prior to this period. As the patient’s travel distance and the GP’s diagnostic strategy and satisfaction level are unknown for this group, it is not straightforward to predict the potential bias. We believe that the group affected by this potential bias is minimal, as previous work has found that 75% of all cancer patients are diagnosed within 73 days of first referral [32].

In cases where the patient is referred to a CPP including a filter function (e.g. gynaecological CPPs), the referral is organised in a central administration and the GP may not know which hospital or practice the patient is referred to (13% of the population). Hence, as the patient’s travel distance is unknown to the GP, the diagnostic strategy cannot be influenced by this knowledge. This is most likely to occur in larger cities where several e.g. gynaecologists practice medicine, and the GP therefore do not know which of the specialised practices the patient would be referred to by the central administration. However, the patient’s distance would most likely not vary substantial since most specialised practices is located rather central. Further, a sensitivity analysis indicated similar results when we excluded cases often diagnosed through a CPP using filter functions (gynaecological cancer, ear-nose-throat cancer, eye cancer or malignant melanoma) *(data not shown).*

The study did not distinguish between which type of referral the GP applied (e.g. CPP, imaginal diagnostic at the hospital etc.), but classified “referral for further diagnostics on the first day” as one. This was chosen due to considerations about sample size and because the available diagnostic investigations vary somewhat across the regions and hospitals in Denmark. Thus, not all GPs have direct access to CT scan and data was not available on which investigations each GP had access too. It could have provided further insights if this data was available, as the GPs perception on access to available investigations and waiting time hereof is likely to reflect their reality and this could therefore potentially influence the time to diagnosis. All GPs can however refer to CPP’s, and it could further be interesting to assess if distance modified the use of CPP.

We adjusted for cancer type as a proxy of the underlying symptom(s) presented by the patient, as this information was not available in the data. However, this approach could be linked with insecurity as symptoms vary within each cancer type.

High celling effect was seen for some of the responses, and few responses were available in some of the categories. Consequently, type II errors cannot be ruled out (e.g. in the stratified analysis on ‘wait-and-see’ and ‘medical treatment’). This could explain why the findings did not reach statistical significance for all categories in these analyses. Consequently, the results should be interpreted with caution and larger-scale studies are required to establish an association. However, to our knowledge no other studies have been carried out to study how distance is associated with the GP’s diagnostic strategies and this study indicate that GP’s of patient with longer distance to further cancer diagnostics more often use “wait-and-se” and “medical treatment” if cancer or serious illness is not the first suspicion of the GP.

### Interpretation

Previous studies have investigated factors of importance for the GP’s propensity to refer patients for cancer diagnostics. The literature suggests that important factors for the GP’s choice of action at first consultation include organisational factors, such as funding system and access to investigations, and contextual factors, such as symptomology and relationship with specialist colleagues [5, 19, 33]. Thus, the diagnostic strategy of the GP, including direct referral or wait-and-see, appears to be multifaceted and to depend on the specific healthcare system, as suggested by a recent study [5].

A study from 2018 combining results on GP referral modalities in 20 countries found that the GPs in 56% of the responding countries agreed that their patients often had to travel long distances to consult a medical specialist [5], but it remains unclear how increasing travel distance to specialist medical care may affect cancer outcomes [6–13].

Few studies have explored underlying factors behind the potential association between travel distance and cancer outcome [15, 16]. To our knowledge, this is the first study to investigate how the patient’s travel distance to the first diagnostic facility influences the GP’s diagnostic strategy. Thereby, it is also one of the first studies attempting to investigate how the patient’s travel distance and related underlying mechanisms may influence cancer outcome. When stratifying the analysis on the GP’s suspicion of cancer, we found an insignificant tendency that increasing travel distance for the patient was associated with increasing use of ‘wait-and-see’ and ‘medical treatment’ when the GP did not suspect cancer. It is a positive factor that the GP’s diagnostic strategy was not influenced by patient’s travel distance when the GP *did* suspect cancer. However, although the results were ambiguous, the insignificant tendency for GPs to use ‘wait-and-see’ for patients with increasing distance to cancer diagnostic facilities indicates that GPs may indeed be affected by the distance to a relevant cancer facility. As all of these patients did turn out to have cancer, this finding should be elaborated further in larger studies.

Increasing travel distance to the patient’s first diagnostic facility was associated with higher GP dissatisfaction with the waiting time for requested diagnostic investigations. This was only found for GPs with patients having more than 49 km between their residential address and the medical facility performing the diagnostic investigation; this could suggest signs of barriers in general practices remotely located from large hospitals. A recent study among GPs in 20 European countries found that rural GPs perceive lower access to diagnostic modalities and the GP perceived more patient related barriers for specialist accessibility [34] which support the findings of dissatisfaction among GPs with increased distance to specialised diagnostics. This dissatisfaction might be rooted in communication barriers between primary and secondary care. Previous studies have pointed to the importance of well-established communication between primary and secondary care for optimal diagnostics [35–37]. As the GP answered the question on satisfaction based on the availability of services in their own practice, this could explain the high rate of dissatisfaction among GPs with long distance who according to the results also tended to be more likely to manage their patients with a wait-and-see strategy if cancer was not suspected. Thus, this could be patients with no alarming symptoms whose GP has the perception of low availability of services, i.e. distant services, which are also distant from the patient’s residence. However, further studies are needed to explore if the findings in the present study are linked to communication barriers or other factors affecting the GP’s perception of waiting time. These studies should also take into account the variability in referral options for the GPs. E.g. not all Danish GPs have the possibility to refer directly to CT scan. It is unknown how this affect the GP’s assessment of available investigations.

## Conclusion

This study found an insignificant tendency for GPs to use ‘wait and see’ and ‘medical treatment’ as diagnostic strategy when cancer or serious illness was not suspected in patients with long travel distance to the medical facility performing the first diagnostic investigation. Longer distance was associated with higher GP dissatisfaction with the waiting time for diagnostic investigations. Further studies with larger samples are required to establish the association.

## Data Availability

Data used for this study is not public available according to Danish laws.

## Abbreviations

CI: Confidence interval
CPP: Cancer patient pathway
CRN: Civil registration number
GDPR: General Data Protection Regulation
GLM: Generalised linear models
GP: General practitioner
ICD-10: International Classification of Diseases, 10th revision
NHISR: (Danish) National Health Insurance Service Register
NPR: (Danish) National Patient Register
PR: Prevalence ratio
SEP: Socio-economic position
